# Supplementary material for: Prevalence of Plasmid-Associated Tetracycline Resistance Genes in Multidrug-Resistant Escherichia coli Strains Isolated from Environmental, Animal and Human Samples in Panama
Source: Antibiotics (Basel). 2023 Jan 31;12(2):280. doi: 10.3390/antibiotics12020280 (PMC9952377; doi:10.3390/antibiotics12020280)
Supplement: Supplementary file 1 [file antibiotics-12-00280-s001.zip › antibiotics-2112073-supplementary.pdf]

antibiotics MDPI

Supplementary filel

Table S1. Multi-drug resistant strains detected.

Site	Sampling source				
	Chicken	Cow	Human	Pig	Water
Ciudad del Niño	10	4	1	8	2
El Arado	8	3	0	7	5

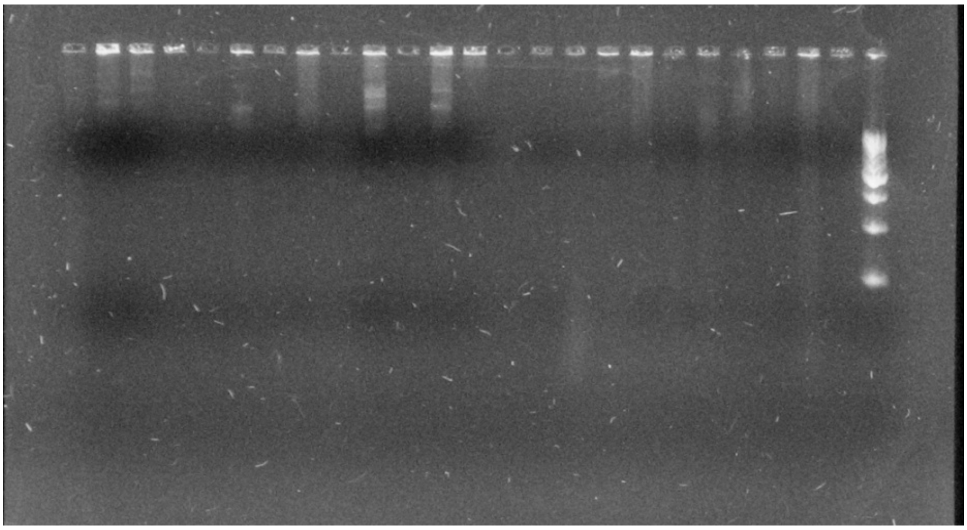

Figure S1. Detection of plasmid DNA by agarose gel electrophoresis. Chemical methods were used to extract and purify plasmid DNA from O/N cultures. Those samples presenting the characteristic bands of plasmid DNA were further analyzed.
